# Levonorgestrel-releasing IUD versus copper IUD in control of dysmenorrhea, satisfaction and quality of life in women using IUD

**Published:** 2012-01

**Authors:** Fatemeh Ramazanzadeh, Toktam Tavakolianfar, Mamak Shariat, Seyed Javad Purafzali Firuzabadi, Fedieh Hagholahi

**Affiliations:** 1 Vali-e-Asr Reproductive Health Research Center, Tehran University of Medical Sciences, Tehran, Iran.; 2 Department of Midwifery, Tarbiat Modaress University, Tehran, Iran.; 3 Maternal, Fetal and Neonatal Research Center, Tehran University of Medical Sciences, Tehran, Iran.; 4 Student Research Assembly, Mashhad University of Medical Sciences, Mashhad, Iran.

**Keywords:** *Dysmenorrhea*, *Intrauterine devices*, *Copper*, *Levonorgestrel-releasing IUD*, *Patient satisfaction*

## Abstract

**Background: **The levonorgestrel-releasing IUD can help the treatment of dysmenorrhea by reducing the synthesis of endometrial prostaglandins as a conventional treatment.

**Objective:** This study was performed to assess the frequency of dysmenorrhea, satisfaction and quality of life in women using Mirena IUDs as compared to those using copper IUDs.

**Materials and Methods:** This double-blind randomized clinical trial was performed between 2006 and 2007 on 160 women aged between 20 to 35 years who attended Shahid Ayat Health Center of Tehran, and they were clients using IUDs for contraception. 80 individuals in group A received Mirena IUD and 80 individuals in group B received copper (380-A) IUD. Demographic data, assessment of dysmenorrhea, and follow-up 1, 3 and 6 months after IUD replacement were recorded in questionnaires designed for this purpose. To assess the quality of life, SF36 questionnaire was answered by the attending groups, and to assess satisfaction, a test with 3 questions was answered by clients.

**Results:** Dysmenorrhea significantly was decreased in both groups six months after IUD insertion as compared to the first month (p<0.001). However, statistically, Mirena reduced dysmenorrhea faster and earlier compared to cupper IUD (p<0.003). There isn’t any significant difference between these two groups in satisfaction and quality of life outcomes.

**Conclusion:** There is no difference between these two groups in terms of the satisfaction and quality of life, therefor the usage of Mirena IUD is not a preferred contraception method.

## Introduction

Dysmenorrhea or painful menstruation is a common gynecological problem which is experienced by 40-70% of all women during their fertile life, 5-20% of whom experience severe pain which reduces their ability in participating in routine activities (1). Some studies have shown that 10-24% of women who suffered from dysmenorrhea asserted that symptoms interfered with their usual activities (2). 

Fifty one percent of those who experienced dysmenorrhea symptoms expressed that these symptoms prevent them from taking apart in their job or their school. Only 31% of these women have reported their symptoms to their physician. In spite of an unusual negative influence on women's quality of life, they do not often go for any medications (2). The conventional treatment of primary dysmenorrhea is based on the prevention of prostaglandin production using non-steroid anti-inflammatory drugs or by preventing ovulation and reducing prostaglandin levels by the use of oral contraceptive pills (3). Copper IUD is a conventional contraceptive method but due to the complications such as dysmenorrhea and hyper menorrhea, 15%- 30% of women request for its removal (4). 

The levonorgestrel-releasing IUD has been successfully used in primary and secondary dysmenorrhea by suppressing endometrial prostaglandin synthesis (5). In addition, the levonorgestrel IUD is especially effective in treating dysmenorrhea caused by recto vaginal endometriosis, which is associated with severe dysmenorrhea, dysparunia and pelvic pain (6). The mechanism of levonorgestrel-releasing IUD is to decrease pelvic pain by releasing 20 µg progestrone locally in the uterine cavity every day, which is more effective than oral therapies (7). 

In addition to its therapeutic role in decreasing dysmenorrhea and severe bleeding during menstruation, the levonorgestrel IUD is also used as a means of contraception (8). The therapeutic effects of levonorgestrel-releasing IUDs on dysmenorrhea, menorrhagia, and contraception have been confirmed in different clinical trials in other countries (7). We decided to do this research because there was no research in terms of comparison between these two IUDs (copper 380 A and Mirena) in Iran.

The main purpose of this study was to assess the frequency of dysmenorrhea and the secondary aim was to evaluate quality of life and satisfaction in women using copper IUDs as compared to levonorgestrel-releasing IUDs in Iranian women.

## Materials and methods

This double blind randomized clinical trial, due to the fact that the individuals under the research and the researcher were both unaware of the type of the IUD and only the one who inserted the IUD has known the type of IUD, was performed on 160 women aged between 20-35 years, who were willing to use IUDs for contraception and had the inclusion criteria for the study. 

This study has been financially supported by Tehran University of Medical Sciences (TUMS). Both IUDs and all cares were free. It has been also approved by the RCT- Scientific and Ethical Committee; Research Deputy of Tehran University of Medical Sciences according to the Helsinki Declaration, clinical trial registration code was 1539/2004.

At the beginning, the aims of study were clarified for the subjects and written consent was obtained. The number of samples was considered based on the below formula. 

$n=\frac{2{\left(Z_{1-\frac{a}{2}}+Z_{1-\beta }\right)}^{2\bar{p}\bar{q}}}{{\left(\mathrm{p1}-\mathrm{p2}\right)}^{2}}$

The existence of dysmenorrhea in cupper IUD based on the previous studies is considered to be 80% and, in the existence of irena is assumed to be 20%, the level of significance and power of study is 95% and 80% respectively. Therefore the sample size in each group was considered 80 cases.

Subjects were divided into two equal groups of 80 patients using Block Randomization method (using 2 to 4 square blocks). Group A received levonorgetrol IUD and Group B received copper 380-A IUD. The levonorgetrol IUD (Mirena, Shering Company, Germany) and the copper IUD produced by Indian companies were inserted in the uterine cavity under sterile conditions by a single gynecologist. 

Regarding the fact that the copper IUD manufactured in India is routinely used in the health centers affiliated to the Iranian Ministry of Health, we considered group B as the control group and group A as the intervention group. The inclusion criteria were, having dysmenorrhea (light, moderate, severe), age (20-35 years), normal menstrual bleeding, the records of one or more pregnancies and normal pop smear in the last 6 months. 

The exclusion criteria included: irregular menstruation, pregnancy, confirmed or suspected cases with malignancy of the uterus, ovaries or breasts, history of breast tumors in patient or close relatives, uterine anomalies, uterine myoma, history of behavioral psychological problems, chronic systemic diseases, PID or history of PID, medical contradiction for hormonal therapy, multi-partnership of the individual or her spouse and history of the use of cardio-vascular drugs before IUD insertion. The patient's history was taken into consideration and pelvic examination was performed. Data were recorded in four questionnaires designed for this purpose: one for demographic data, second for gynecological and menstrual history, which were completed by a trained midwife. The third one was to assess the quality of life (SF 36) and the last one was to determine satisfaction, and they were completed by the clients. SF36 Questionnaire evaluates the mental and physical health, and it has been translated to Persian by Montazeri )^9^). Satisfaction questionnaire includes 3 questions:

1) Are you satisfied with the IUD you use?

2) Do you prefer to keep using this device?

3) Do you want to suggest these IUDs to the others? 

Dysmenorrhea, as the main outcome of this study, was assessed by using a self- expression questionnaire prior to IUD insertion as well as in the follow- up visits classified scales: no reduction, increase, and no change, were completely alleviated as compared to the period before IUD insertion. Each volunteer was compared with not only the other group but with herself (before and after IUD insertion). All women had dysmenorrheal before IUD insertion. SPSS version 11.5 computer software was used for data entry and analysis. Descriptive statistical analysis was in the form of relative and absolute frequencies for qualitative variables, while mean and standard deviation were used for quantitative variables. 

**Statistical analysis**

Analytical data were extracted using chi-square, student’s t- test, and repeated measure analysis for the comparison of intrinsic changes between the two groups. The power of the study was 80% and confidence interval was considered as 95%.

## Results

This study was performed on 160 women between the ages of 20-35 years old with mean age of 26.54±4.25 years in group A and 26.49±4.37 years in group B. The mean for the duration of the 

menstruation in group A was 6.8±1.8 days and in group B is 6.11±1.6 days. In table I the demographic and midwifery characteristics in two groups are compared. Results show that a significant statistical difference did not exist between the two groups regarding background variables and confining factors, which may affect the outcome of the study. 

Graph 1 showed change in dysmenorrhea during the six- months' interval, and it is seen that in both groups, dysmenorrhea significantly decreased after 6 months. Levonorgestrel-releasing IUD significantly decreases dysmenorrhea earlier and more considerable as compared to copper IUD (p<0.003). 1, 3 and 6 months after the insertion of IUDs the frequency of dysmenorrheal in cupper A was 36.7, 43.2 and 8.6 percent respectively, while in the mentioned time the frequency of dysmenorrhea in Mirena was 18.8, 6.3 and 2.7 percent, respectively. 

The frequency of dysmenorrhea was not statistically different in the first and six months in either of the two groups after IUDs insertion; however, it was significantly increased in the third month among women using copper-T 380-A as compared to those using levonorgestrel-IUD (p<0.01). In Mirena using group, 64%, and, in Copper T using group, 68.6% of the tested women were satisfied, which were not significant. Table II shows that there was no significant difference between the quality of life (36 score) in 2 groups at the beginning of the research (2 groups were equal). Table III shows the quality of life in both groups after and before IUDs insertion. 

The quality of life in Mirena group and Copper T users increased dramatically in comparison to the first visit except for the General health. (The general health remains the same.) (p-values are shown in tables).

**Table I:** Comparison of background and demographic factors in the two groups

**Demographic and obstetric characteristics**		**Group A** **(Levonorgestrel IUD)**	**Group B** **(Copper IUD)**	**p-value**
Age (years) mean ±SD		4.25±26.54	4.3±26.49	0.91 (Ns)
Level of education				
	Below high-school diploma	26 (3.5%)	26 (32.5%)	0.5 (Ns)
	Above high-school diploma	54 (67.5%)	30 (37.5%)	
Occupation				
	Housewife	75 (93.8%)	76 (95%)	0.7 (Ns)
	Working	5 (6.3%)	4 (5%)	
Duration of menstruation (days)		1.8±6.8	1.6±6.11	0.7 (Ns)
No. of pregnancies		0.6±1.5	0.9±1.77	0.2 (Ns)
No. of deliveries		0.8±1.68	1.5±1.85	0.3 (Ns)
Duration since last delivery				
	Less than 12 months	55 (68%)	44 (34%)	0.1 (Ns)
	More than 12 months	25 (31.3%)	57 (42.5%)	

**Table II:** Comparing the quality of life between 2 groups at the beginning of the research

**Index**	**Mirena IUD**		**Copper IUD**	
	**Standard deviation**	**Mean**	**Standard deviation**	**Mean**
Physical function	38.84	71.42	35.83	84.78
Role-physical	28.02	75.31	24.17	81.44
Bodily pain	22.45	69	25.70	64.84
General health	16.69	75.23	18.08	70.98
Vitality	15.91	63.73	14.58	63.17
Social function	20.84	85.51	23.72	84.37
Role-emotional	38.40	73.54	41.36	69.87
Mental health	14.56	69.96	15.45	68.92

**Table III:** Comparing the quality of life in Mirena users before and after insertion

**Index**	**Copper**		**p-value**	**Mirena**		**p-value**	**p-value** **(Between two groups)**
	**(Mean±SD)**			**(Mean±SD)**			
	**Before**	**After**		**Before**	**After**		
Physical function	**81.44**	99.03	<0.0001	75.31	96.82	<0.0001	0.34
Role-physical	7.15	100	<0.0001	6.85	100.0	<0.0001	
Bodily pain	64.84	84.65	<0.0001	69.00	85.88	<0.0001	0.62
General health	70.98	72.9	<0.738	75.23	74.58	<0.738	0.25
Vitality	84.37	**97.35**	<0.028	63.73	68.73	<0.028	0.49
Social gunction	84.37	97.35	<0.0001	85.51	96.82	<0.0001	0.83
Role-emotional	5.09	98.07	<0.0001	5.20	100.00	<0.0001	0.96
Mental health	68.92	75.46	<0.018	69.96	74.22	<0.052	0.56

**Figure 1:**
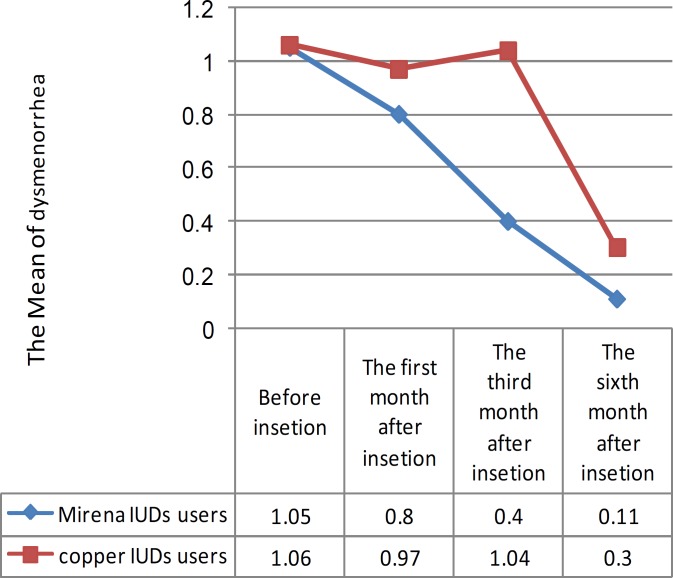
The changes of dysmenorrhea´s mean during six month in copper IUDs users and Mirena IUDs users

This graph shows the dysmenorrhea in two groups at the beginning of the study, 1, 3 and 6 months after IUD insertion, that was according to the result of repeated measure analysis of variance.

In this analysis dysmenorrheal, two IUDs and time were considered as main variable, between subject factor and within subject factor, respectively.

## Discussion

The accumulations of prostaglandins (prostaglandin E_2_, prostaglandin F2α) are significantly higher in primary dysmenorrhea as compared to women who do not suffer from dysmenorrhea (9, 10). Therefore, non- steroid anti-inflammatory drugs are considered to be effective in the treatment of primary and secondary dysmenorrhea by suppressing prostaglandin synthesis (11). By releasing a daily dose of 20µg progesterone and by its effect on the endometrium, levonorgestrel-releasing IUD can treat many gynecological problems as well as to provide a good means of contraception (11, 12). During the 6 month period in which levonorgestrel-releasing IUDs were used, dysmenorrhea decreased significantly, not only as compared to copper IUD users, but also as compared to the period before IUD insertion. 

Many studies have been performed in other countries regarding the effect of levonorgestrel- releasing IUD on menorrhagia and dysmenorrhea, the efficacy of which has been confirmed in reducing menstrual bleeding and dysmenorrhea (11). In the study performed by Wid meersch *et al* (2001), 12 women were followed up for at least 12 months, pain was reported to decrease in all cases markedly, with complete elimination of dysmenorrhea in some cases (13).

Sheng *et al* (2009) showed the considerable reduction of pain and endometriosis during a 36 month period of using levonorgestrel-IUD in 94 Chinese women (14). In a review article by Regine and Pinjo (2005), levonorgestrel-IUD was studied as a means of contraception not only with therapeutic effects, but also with ability to reduce dysmenorrhea around 80% (15). In other review studies, Gupta *et al* (2007) in India, Bahamondes *et al* (2008) in Brazil, ESHRE in Italy (2008), Kriplani *et al*, in India (2007), and Mansour, in England (2008) also pointed out the reduction in dysmenorrhea and menorrhagia during the course of levonorgestrel- IUD use (16, 20). 

Jimenez *et al* in Brazil (2008) also stressed on the reduction in uterine blood flow and endometrial thickness after using levonorgestrel- IUD as compared to the copper IUD and the direct relationship between uterine blood flow reduction and reduced dysmenorrhea (21, 22). Considering the similar results of the present study, in regard to the satisfaction rate with using the pregnancy preventive devices and their effect on the quality of life, which are among the important factors, we can determine which method should be chosen, and considering the satisfaction rate in the two groups, although they do not show any significant difference. 

Due to high price of Mirena IUD it is advised to use Copper IUD, but not as a means of prevention for all Iranian women. The use of Mirena IUD is recommended to women as a treatment, whenever they suffer from dysmenorrhea in the period of menstruation. Moreover, it can be useful when they are sensitive to the use of Copper IUD. Finally we could point out the effective role of levonorgestrel- IUD in reducing dysmenorrhea. This kind of IUD could be recommended for use by women, as the treatment of dysmenorrhea.

## Footnote

***Registration ID in IRCT: IRCT138903013881N2***
